# Ambulatory blood pressure levels in individuals with uncontrolled clinic hypertension across Bangladesh, Pakistan, and Sri Lanka

**DOI:** 10.1111/jch.14787

**Published:** 2024-03-07

**Authors:** Anqi Zhu, Truls Ostbye, Aliya Naheed, H Asita de Silva, Imtiaz Jehan, Mihir Gandhi, Nantu Chakma, Anuradhani Kasturiratne, Zainab Samad, Tazeen Hasan Jafar

**Affiliations:** ^1^ Program in Health Services & Systems Research Duke‐NUS Medical School Singapore Singapore; ^2^ Duke University Department of Family Medicine and Community Health Duke University Medical Center Durham North Carolina USA; ^3^ Health Systems and Population Studies Division International Centre for Diarrhoeal Disease Research (ICDDR, B) Dhaka Bangladesh; ^4^ Department of Pharmacology Faculty of Medicine University of Kelaniya Ragama Sri Lanka; ^5^ Department of Community Health Science Aga Khan University Karachi Pakistan; ^6^ Biostatistics Singapore Clinical Research Institute Singapore Singapore; ^7^ Centre of Quantitative Medicine Duke‐NUS Medical School Singapore Singapore; ^8^ Tampere Center for Child Health Research Tampere University Tampere Finland; ^9^ Department of Public Health Faculty of Medicine University of Kelaniya Ragama Sri Lanka; ^10^ Department of Medicine Medical College Aga Khan University Karachi Pakistan; ^11^ Duke Global Health Institute Durham North Carolina USA

**Keywords:** ambulatory blood pressure monitor, South Asia, uncontrolled clinic hypertension

## Abstract

Hypertension is a leading risk factor for cardiovascular disease in South Asia. The authors aimed to assess the cross‐country differences in 24‐h ambulatory, daytime, and nighttime systolic blood pressure (SBP) among rural population with uncontrolled clinic hypertension in Bangladesh, Pakistan, and Sri Lanka. The authors studied patients with uncontrolled clinic hypertension (clinic BP ≥ 140/90 mmHg) who underwent ambulatory blood pressure monitoring (ABPM) during the baseline assessment as part of a community‐based trial. The authors compared the distribution of ABPM profiles of patients across the three countries, specifically evaluating ambulatory SBP levels with multivariable models that adjusted for patient characteristics. Among the 382 patients (mean age, 58.3 years; 64.7% women), 56.5% exhibited ambulatory hypertension (24‐h ambulatory BP ≥ 130/80 mmHg), with wide variation across countries: 72.6% (Bangladesh), 50.0% (Pakistan), and 51.0% (Sri Lanka; *P *< .05). Compared to Sri Lanka, adjusted mean 24‐h ambulatory, daytime, and nighttime SBP were higher by 12.24 mmHg (95% CI 4.28–20.20), 11.96 mmHg (3.87–20.06), and 12.76 mmHg (4.51–21.01) in Bangladesh, separately. However, no significant differences were observed between Pakistan and Sri Lanka (*P *> .05). Additionally, clinic SBP was significantly associated with 24‐h ambulatory (mean 0.38, 95% CI 0.28–0.47), daytime (0.37, 0.27–0.47), and nighttime SBP (0.40, 0.29–0.50) per 1 mmHg increase. The authors observed substantial cross‐country differences in the distribution of ABPM profiles among patients with uncontrolled clinic hypertension in rural South Asia. The authors findings indicated the need to incorporate 24‐h BP monitoring to mitigate cardiovascular risk, particularly in Bangladesh.

## 1 INTRODUCTION

Elevated blood pressure (BP), affecting over 27% of South Asian adults, is a leading modifiable risk factor contributing to the burden of cardiovascular disease (CVD) and premature death in the region.^1^, ^2^ This is especially concerning, given that the South Asian population is known to have higher risks of stroke and coronary heart disease than other ethnic groups.^3^, ^4^ Besides, the predominantly low‐ and middle‐income status of most South Asian countries poses a substantial challenge in managing CVD, particularly in rural areas where healthcare systems are resourced‐limited.^3^

In 2019, stroke accounts for around 29% of the increasing CVD disability‐adjusted life years (DALYs) in South Asia, with Bangladesh experiencing the highest age‐standardized DALYs for stroke, surpassing Pakistan and Sri Lanka (Figure S1).^5^ One potential reason for such disparity could be variations in BP levels. Isolated BP measurements taken during clinic visits could not accurately reflect an individual's BP level.^6^, ^7^ Recent hypertension guidelines have thus recommended more extensive use of ambulatory blood pressure monitoring (ABPM) to improve CVD risk management in patients diagnosed with clinic hypertension,^8^ which automatically records BP at regular intervals for 24 h.^9^ The generated ABPM profile includes components such as 24‐h ambulatory, daytime, and nighttime systolic blood pressure (SBP), which studies have consistently demonstrated to be better predictors of target organ damage, cardiovascular events, and overall mortality compared to clinic BP.^10^, ^11^, ^12^ Studies of South Asians living in the United Kingdom showed important differences in clinic BP levels by country of origin.^13^, ^14^, ^15^ The Newcastle Heart Project study and the 1999 Health Survey for England showed that Bangladeshi men and women had lower age‐standardized mean clinic BP than their Indian and Pakistani counterparts.^14^, ^15^ However, it remains unknown whether cross‐country differences in ambulatory BP profiles exist among their compatriots still living in South Asia.

Our study, leveraging the baseline data collected from the Control of Blood Pressure and Risk Attenuation (COBRA) trial, aimed to assess the cross‐country differences in 24‐h ambulatory, daytime, and nighttime SBP levels among rural populations with uncontrolled clinic hypertension (treated or untreated) in Bangladesh, Pakistan, and Sri Lanka. Given the observed cross‐country disparities in stroke burden, we hypothesized significant differences in the distribution of 24‐h ambulatory, daytime, and nighttime SBP among patients across the three countries. Additionally, we aimed to determine the proportion of patients with ambulatory hypertension in each country, and to explore the potential associations of patient characteristics (including clinic BP) with ambulatory SBP levels.

## 2 METHODOLOGY

### 2.1 Study design, setting, and patient population

This multicountry study was embedded in the COBRA cluster‐randomized controlled trial in Bangladesh, Pakistan, and Sri Lanka.^16^

The COBRA trial was conducted in rural settings across multiple districts, including Tangail and Munshigaonj in Bangladesh, Thatta in Pakistan, and Putlam in Sri Lanka. A total of 30 rural clusters were randomly selected, each having 250−300 households. These clusters were grouped into geographically contiguous administrative units served by government clinics. Ten units were first randomly selected from each country, followed by a random selection of one cluster per unit based on their distance from the clinic. Details of the study design have been described elsewhere.^16^

Briefly, the recruitment of trial patients involved research staff visiting all adults aged 40 years or older in the selected clusters based on information provided by the Local Health Office. Individuals who consented were screened for eligibility, including measuring clinic BP using the calibrated Omron HEM‐7300 Blood Pressure Monitor (Omron Corporation, Japan) in a sitting position. Readings were taken three times with at least 3‐min intervals, then the mean of the last two measurements was recorded. Individuals with elevated clinic BP readings (clinic BP ≥ 140/90 mmHg) were revisited after 2 weeks to confirm their hypertension status, while those already on antihypertensive medications were recruited during the first visit.

For this study, patients selected for 24‐h ABPM tests during the trial were included. Patients with consistently elevated clinic BP readings in each set of two readings from two separate days would be eligible for ABPM tests. A total of 420 patients were recruited (14 patients per cluster) to undergo ABPM tests at the baseline, year 1, and year 2 of the trial. The data collection timeframe for the baseline ABPM tests spanned from July 2016 to Sept 2017, with minor variations across the three countries. Specifically, we utilized the data collected at the baseline of the trial for our analysis primarily due to its capacity to mitigate potential bias stemming from the antihypertensive intervention's impact on ABPM data.

### 2.2 ABPM measurements

Research staff responsible for placing the ABPM monitors underwent training to ensure consistency and accuracy. The training included a workshop facilitated by an ABPM specialist, which focused on familiarizing the research staff with the ABPM equipment, calibration procedures, and the normal ranges of ABPM measurements. Additionally, they were provided with a standard operating procedure and a clinic checklist to ensure proper fitting of the ABPM monitors.

Before the ABPM tests, precautions were taken to confirm that patients were sufficiently capable and comfortable in wearing the ABPM monitors. Then, patients were invited to a designated facility within their cluster, with free transportation provided. Trained research staff would apply the validated Spacelabs 90207 model (Spacelabs Healthcare, US) on the nondominant arm of each patient to conduct the ABPM tests. Following the application of ABPM monitors, the patients were given standardized instructions about using the monitor. Monitoring intervals were set at 30‐min intervals during the day (07:00–22:00) and hourly intervals during the night (22:00–07:00). However, for patients who worked night shifts, the monitoring intervals were adjusted accordingly to accommodate their specific circumstances. After 24 h, patients returned to the facilities for monitor removal. In accordance with the ABPM guideline,^9^ ABPM measurements with at least 70% valid readings, including at least 14 valid daytime readings and 7 valid nighttime readings, were considered acceptable.

### 2.3 Statistical analysis

#### 2.3.1 Outcomes

We focused on three primary outcomes: 24‐h ambulatory SBP, daytime SBP, and nighttime SBP levels. The 24‐h ambulatory SBP represented the average SBP over 24‐h, while daytime and nighttime SBP were defined as the mean of SBP readings measured during the programmed day/night period. Our secondary outcome was ambulatory hypertension, which was defined as 24‐h ambulatory SBP ≥ 130 mmHg and/or 24‐h ambulatory diastolic blood pressure (DBP) ≥ 80 mmHg, each representing the mean ambulatory BP values over 24 h.^17^ Additionally, we explored other components of the ABPM profile, including ambulatory DBP levels, abnormal nocturnal dipping (defined as night/day SBP ratio > 0.9), and 24‐h cumulative BP load.^9^ While the clinical significance of BP load remains unclear, recent findings suggest that the 24‐h cumulative BP load, calculated as the area under the curve (AUC) between the ambulatory BP curve and time axis using the composite trapezoid rule, is predictive of target organ damage.^18^ Consequently, we have adopted this component in our study.

#### 2.3.2 Analysis

First, the distributions of individual patient characteristics and ABPM profiles were compared across Bangladesh, Pakistan, and Sri Lanka. The *P*‐values were calculated by adjusting for cluster‐specific random intercepts in Generalized Linear Mixed Model (GLMM) to account for potential clustering effect within the data which comprised 30 clusters. Then, we employed GLMM to examine the associations between country and 24‐h ambulatory SBP while adjusting for potential covariates, including age (per 1 year), sex (female/male), clinic SBP (per 1 mmHg), education status (no formal education/educated), socioeconomic levels (poor, middle, rich), marital status (currently unmarried/married), employment status (currently unemployed/employed), obesity/overweight (yes/no), smoking habits (never smoke, former smoker, current smoker), frequency of fruit and vegetable intake per week (per 1 unit increase), physical activity level (inactive, minimally active, highly active), chronic kidney disease (CKD, yes/no), diabetes (yes/no), the number of antihypertensive medications used (0, 1, 2, ≥ 3), statins usage (currently using statins/currently not on statins), high‐density lipoprotein cholesterol (HDL, per 10 mg/dL increase), low‐density lipoprotein cholesterol (LDL, per 10 mg/dL increase), and triglycerides levels (per 10 mg/dL increase). Including CKD and diabetes mellitus in the model was justified by previous studies, which showed an increased risk of elevated ambulatory BP levels and altered circadian BP rhythm in the presence of these comorbidities.^19^, ^20^ Details about the measurement and definitions of these covariates can be found in Supplemental information.

We also adjusted for the same set of potential covariates to examine the association between country and both daytime and nighttime SBP levels. For each model, generalized variance inflation factor (GVIF) was used to detect multicollinearity so that highly correlated patient characteristics (GVIF > 10) could be removed.^21^ Cluster‐specific random intercepts were also included to account for potential clustering effect. Standardized coefficients were calculated after standardizing all continuous variables, including both the dependent and independent variables, to z‐scores using the mean and standard deviation (SD).

Sensitivity analyses were conducted to examine the robustness of our results. Since we applied multiple imputations for missing patient covariates with country as the level‐2 variable, we performed the analysis on the subset of complete cases for comparison. The distribution of the missing covariates did not differ substantially between patients with observed data and those with imputed data (Table S1). We also evaluated the sensitivity to departures to missing‐at‐random (MAR) assumption with the delta‐adjustment method. The impact of multiple imputation was thus explored by allowing the degree of departure (delta) to MAR to vary by 0%, 10%, 20%, and 50% of the means of observed data in all continuous missing patient covariates simultaneously. Further, we stratified the multivariable models by country to explore the potential effect modification and identify any significant interaction terms between patient characteristics and country. A *P‐*value threshold of <.05 was considered statistically significant. All the analyses were performed using R version 4.2.1.

## 3 RESULTS

A total of 420 patients aged 40 years or older with uncontrolled clinic hypertension (clinic BP ≥ 140/90 mmHg) were initially recruited from Bangladesh, Pakistan, and Sri Lanka to undergo ABPM tests at the baseline of the COBRA trial. Among them, 382 patients provided ABPM measurements that met the acceptability criteria outlined in the ABPM guideline,^9^ and their data were included in this study and constituted the final analysis sample.

### 3.1 Distribution of individual patient characteristics

Table 1 presents the characteristics of the 382 patients with uncontrolled clinic hypertension across the three countries: 106 in Bangladesh, 178 in Pakistan, and 98 in Sri Lanka.

**TABLE 1:** Sociodemographic and disease characteristics of patients with uncontrolled clinic hypertension across rural Bangladesh, Pakistan, and Sri Lanka.

	Bangladesh (N = 106)	Pakistan (N = 178)	Sri Lanka (N = 98)	Total (N = 382)	P‐value^*^
**Sociodemographic variables**					
Age in year, mean (SD)	58.0 (11.3)	56.4 (11.1)	62.1 (9.8)	58.3 (11.0)	.003
Sex, n (%)					.299
Female	73 (68.9%)	107 (60.1%)	67 (68.4%)	247 (64.7%)	
Male	33 (31.1%)	71 (39.9%)	31 (31.6%)	135 (35.3%)	
Marital status, n (%)					.133
Currently unmarried	29 (27.4%)	40 (22.5%)	33 (33.7%)	102 (26.7%)	
Married	77 (72.6%)	138 (77.5%)	65 (66.3%)	280 (73.3%)	
Socioeconomic level, n (%)					.709
Poor	24 (22.6%)	34 (19.1%)	15 (15.3%)	73 (19.1%)	
Middle	71 (67.0%)	125 (70.2%)	68 (69.4%)	264 (69.1%)	
Rich	11 (10.4%)	19 (10.7%)	15 (15.3%)	45 (11.8%)	
Education status, n (%)					<.001
Not received formal education	51 (48.1%)	131 (73.6%)	2 (2.0%)	184 (48.2%)	
Educated	55 (51.9%)	47 (26.4%)	96 (98.0%)	198 (51.8%)	
Employment status, n (%)					.059
Currently unemployed	84 (79.2%)	110 (61.8%)	74 (75.5%)	268 (70.2%)	
Employed	22 (20.8%)	68 (38.2%)	24 (24.5%)	114 (29.8%)	
**Lifestyle variables**					
BMI in kg/m^2^, mean (SD)	23.7 (4.0)	25.0 (5.3)	26.0 (4.6)	24.9 (4.8)	.004
(Missing)	0	0	3	3	
Waist circumference in cm, mean (SD)	84.6 (10.9)	88.6 (12.9)	92.5 (12.1)	88.5 (12.5)	.001
Obese/overweight, n (%) (Ref: Nonobese)	48 (45.3%)	108 (60.7%)	65 (68.4%)	221 (58.3%)	.004
(Missing)	0	0	3	3	
Frequency of fruit and vegetable intake per week, mean (SD)	17.0 (7.7)	6.9 (2.7)	18.2 (7.9)	12.5 (8.0)	<.001
(Missing)	0	0	6	6	
Physical activity level, n (%)					.377
Inactive	24 (22.6%)	68 (38.4%)	23 (23.7%)	115 (30.3%)	
Minimally active	29 (27.4%)	20 (11.3%)	24 (24.7%)	73 (19.2%)	
Highly active	53 (50.0%)	89 (50.3%)	50 (51.5%)	192 (50.5%)	
(Missing)	0	1	1	2	
Smoking habit, n (%)					.169
Never Smoke	84 (79.2%)	130 (73.0%)	80 (81.6%)	294 (77.0%)	
Former Smoker	14 (13.2%)	27 (15.2%)	16 (16.3%)	57 (14.9%)	
Current Smoker	8 (7.5%)	21 (11.8%)	2 (2.0%)	31 (8.1%)	
**Comorbidities**					
Self‐reported heart disease, n (%)	21 (19.8%)	8 (4.5%)	10 (10.2%)	39 (10.2%)	.006
Self‐reported stroke, n (%)	27 (25.5%)	10 (5.6%)	5 (5.1%)	42 (11.0%)	<.001
Chronic kidney disease, n (%)	56 (52.8%)	32 (20.9%)	77 (81.9%)	165 (46.7%)	<.001
(Missing)	0	25	4	29	
Diabetes, n (%)	19 (17.9%)	29 (18.2%)	28 (29.8%)	76 (21.2%)	.068
(Missing)	0	19	4	23	
**Medication usage**					
Number of antihypertensive medications used, n (%)					<.001
0	2 (1.9%)	147 (82.6%)	7 (7.1%)	156 (40.8%)	
1	63 (59.4%)	28 (15.7%)	44 (44.9%)	135 (35.3%)	
2	32 (30.2%)	2 (1.1%)	35 (35.7%)	69 (18.1%)	
3 or more	9 (8.5%)	1 (0.6%)	12 (12.2%)	22 (5.8%)	
Currently using satins (Ref: Not currently on statins), n (%)	7 (6.6%)	4 (2.2%)	41 (41.8%)	52 (13.6%)	<.001
**Clinical variables**					
Clinic SBP in mmHg, mean (SD)	161.0 (16.0)	152.2 (16.4)	163.4 (20.9)	157.5 (18.2)	<.001
Clinic DBP in mmHg, mean (SD)	96.3 (12.2)	93.9 (12.0)	96.0 (13.0)	95.1 (12.3)	.209
eGFR in mL/min per 1.73 m^2^, mean (SD)	75.6 (21.0)	95.5 (19.1)	52.8 (10.8)	78.5 (25.0)	<.001
(Missing)	0	19	4	23	
Log_e_ urine spot albumin‐to‐creatinine ratio in mg/g, mean (SD)	2.9 (1.8)	2.4 (1.2)	3.2 (0.9)	2.8 (1.4)	.001
(Missing)	0	24	4	28	
24‐h urine sodium estimates in g/day, mean (SD)	4.3 (1.5)	4.1 (1.4)	5.4 (1.4)	4.5 (1.5)	<.001
(Missing)	0	24	5	29	
HDL cholesterol in mg/dL, mean (SD)	38.9 (10.9)	42.4 (11.8)	54.4 (9.2)	44.5 (12.4)	<.001
(Missing)	0	20	4	24	
LDL cholesterol in mg/dL, mean (SD)	130.7 (36.7)	111.6 (33.0)	132.1 (41.3)	122.5 (37.6)	.002
(Missing)	2	19	4	25	
Fasting blood glucose in mg/dL, median (IQR)	99.2 (91.1, 118.3)	94.0 (86.0, 110.5)	110.3 (100.0, 134.0)	99.8 (90.0, 119.0)	.001
(Missing)	0	19	4	23	
Triglycerides in mg/dL, mean (SD)	165.7 (78.4)	152.3 (78.3)	128.1 (59.5)	149.9 (75.1)	.005
(Missing)	1	19	5	25	
Total cholesterol in mg/dL, mean (SD)	192.0 (37.5)	175.8 (40.5)	210.9 (48.6)	189.7 (44.2)	<.001
(Missing)	3	19	5	27	

*Notes*: Obese/overweight is defined as BMI ≥ 23.5 kg/m^2^.^40^ Chronic kidney disease is defined as eGFR ≤ 60 mL/min per 1.73 m^2^ and/or urine albumin‐to‐creatinine ratio ≥ 30 mg/g.^41^ Diabetes mellitus is defined as fasting plasma glucose ≥ 126 mg/ml.^42^

Abbreviations: BMI, body mass index; DBP, diastolic blood pressure; eGFR, estimated glomerular filtration rate; HDL, high‐density lipoprotein; IQR, interquartile range; LDL, low‐density lipoprotein; SBP, systolic blood pressure; SD, standard deviation.

* *P*‐values were computed using linear or generalized linear mix‐effect models with cluster‐specific random intercepts to account for the clustering effects within the data.

Overall, the study patients in the three countries had a mean age of 58.3 (SD 11.0) years, and 64.7% of them were women. The distribution of sociodemographic, lifestyle, comorbidities, medication usage, and clinical variables varied across the countries. For example, Sri Lanka had a higher proportion of patients who received formal education (98.0%) compared to Bangladesh (51.9%) and Pakistan (26.4%) (*P *< .001).

Patients in Sri Lanka had the highest mean clinic SBP level of 163.4 mmHg (SD 20.9), while patients in Bangladesh had a mean clinic SBP level of 161.0 mmHg (SD 16.0) and those in Pakistan had a mean level of 152.2 mmHg (SD 16.4) (*P* < .001). (See Figure S2A for pairwise comparison).

The proportion of patients with self‐reported heart disease was highest in Bangladesh (19.8%), followed by Sri Lanka (10.2%) and Pakistan (4.5%) (*P* = .006). Patients in Bangladesh also had the highest proportion of self‐reported strokes (25.5%), while relatively lower proportions were observed in Pakistan (5.6%) and Sri Lanka (5.1%) (*P *< .001). In addition, the highest proportion of patients with CKD was found in Sri Lanka (81.9%), followed by Bangladesh (52.8%) and Pakistan (20.9%) (*P *< .001).

Substantial variations were also observed in the use of antihypertensive medications and statins across the three countries. Bangladesh had the highest proportion of patients taking antihypertensive medications (98.1%), followed by Sri Lanka (92.9%) and Pakistan (17.4%) (*P *< .001).

### 3.2 Comparison of the distribution of ABPM profiles across countries

**FIGURE 1:**
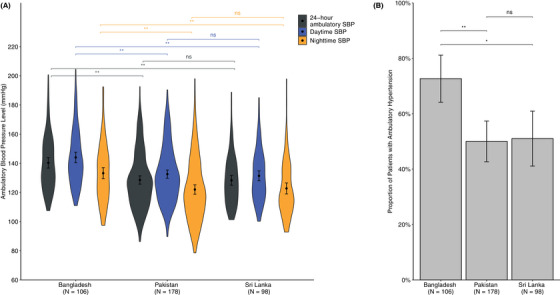
Comparison of ambulatory systolic blood pressure levels and the proportion of individuals with ambulatory hypertension in patients with uncontrolled clinic hypertension across rural Bangladesh, Pakistan, and Sri Lanka. (A) Violin plot comparing the distribution of 24‐h ambulatory, daytime, and nighttime SBP levels in patients with uncontrolled clinic hypertension across the three countries. (B) Bar plot comparing the proportions of patients having ambulatory hypertension (defined as 24‐h ambulatory SBP/DBP ≥ 130/80 mmHg) across the three countries. The black dots represented the mean and the error bars represented 95% confidence interval (CI). Pairwise comparison was conducted using the t‐test, with Sri Lanka serving as the reference group. ns ≥ 0.05, ^*^ *p* < .05, ^**^ *p* < .001.

Among patients with uncontrolled clinic hypertension, we observed statistically significant cross‐country differences in the distribution of 24‐h ambulatory SBP (Table 2). In terms of mean 24‐h ambulatory SBP level, Bangladesh had the highest mean level (140.3 mmHg, SD 18.6), followed by Pakistan (128.6 mmHg, SD 19.4) and Sri Lanka (128.3 mmHg, SD 17.0) (*P *= .003). Similar patterns were observed for daytime and nighttime SBP, with Bangladesh leading in mean daytime SBP (144.0 mmHg, SD 19.0), followed by Sri Lanka (131.4 mmHg, SD 17.1) and Pakistan (132.6 mmHg, SD 19.2) (*P *= .002). Likewise, for nighttime SBP, Bangladesh had the highest level (133.2 mmHg, SD 20.0), followed by Sri Lanka (122.8 mmHg, SD 18.9) and Pakistan (122.0 mmHg, SD 21.8) (*P *= .037). (See Figure 1A for pairwise comparison with consistent results). The distributions of 24‐h ambulatory, daytime, and nighttime SBP levels revealed a different pattern compared to that of the clinical SBP levels across the three countries (Figure S2A).

**TABLE 2:** Distribution of ABPM profiles among patients with uncontrolled clinic hypertension across rural Bangladesh, Pakistan, and Sri Lanka.

ABPM profile	Bangladesh (N = 106)		Pakistan (N = 178)		Sri Lanka (N = 98)			
Mean(SD)/N (%)	95% CI	Mean(SD)/N (%)	95% CI	Mean(SD)/N (%)	95% CI	Mean(SD)/N (%)	Total (N = 382)	P‐value^*^
24‐h ambulatory SBP, mmHg	140.3 (18.6)	(136.7, 143.9)	128.6 (19.4)	(125.7, 131.4)	128.3 (17.0)	(124.9, 131.7)	131.8 (19.3)	.003
24‐h ambulatory DBP, mmHg	79.3 (10.8)	(77.2, 81.4)	78.9 (11.2)	(77.2, 80.5)	74.6 (8.9)	(72.8, 76.4)	77.9 (10.7)	.030
Ambulatory hypertension (Ref: ambulatory normotension)	77 (72.6%)	(63.5%, 80.2%)	89 (50.0%)	(42.7%, 57.3%)	50 (51.0%)	(41.3%, 60.7%)	216 (56.5%)	.014
Daytime SBP, mmHg	144.0 (19.0)	(140.4, 147.7)	132.6 (19.2)	(129.7, 135.4)	131.4 (17.1)	(128.0, 134.8)	135.4 (19.3)	.002
Daytime DBP, mmHg	81.5 (11.7)	(79.2, 83.7)	82.0 (11.2)	(80.3, 83.6)	77.0 (9.3)	(75.2, 78.9)	80.6 (11.1)	.010
Nighttime SBP, mmHg	133.2 (20.0)	(129.4, 137.1)	122.0 (21.8)	(118.8, 125.2)	122.8 (18.9)	(119.0, 126.6)	125.3 (21.1)	.037
Nighttime DBP, mmHg	75.2 (10.5)	(73.2, 77.2)	73.8 (12.3)	(72.0, 75.6)	69.9 (9.3)	(68.1, 71.8)	73.2 (11.3)	.017
Night‐to‐day SBP ratio	0.9 (0.1)	(0.9, 0.9)	0.9 (0.1)	(0.9, 0.9)	0.9 (0.1)	(0.9, 1.0)	0.9 (0.1)	.477
Abnormal nocturnal dipping (Ref: normal nocturnal dipping)	70 (66.0%)	(56.6%, 74.4%)	102 (57.3%)	(50.0%, 64.3%)	63 (64.3%)	(54.4%, 73.1%)	235 (61.5%)	.442
24‐h cumulative SBP load, mmHg x h	3231.1 (431.2)	(3148.0, 3314.1)	2957.8 (437.6)	(2893.0, 3022.5)	2950.2 (382.1)	(2873.6, 3026.8)	3031.7 (439.0)	<.001
24‐h cumulative DBP load, mmHg x h	1827.1 (251.8)	(1778.6, 1875.6)	1816.6 (251.8)	(1779.4, 1853.9)	1718.8 (202.7)	(1678.2, 1759.5)	1794.4 (243.7)	.003

*Notes*: Ambulatory hypertension is defined as 24‐h ambulatory SBP ≥ 130 mmHg and/or 24‐h ambulatory DBP ≥ 80 mmHg.^17^ Daytime SBP and DBP were calculated as the mean of at least 14 valid BP readings obtained during the daytime period (totaling 30 readings, 07:00–22:00), while nighttime SBP and DBP were the mean of at least 7 valid BP readings during nighttime (totaling 9 readings, 22:00–07:00), based on the predefined settings of the ABPM device. Normal nocturnal dipping status referred to night/day SBP ratio > 0.9, whereas a ratio ≤ 0.9 indicated abnormal nocturnal dipping status. The 24‐h cumulative SBP and DBP load were calculated as the area under curve (AUC) between fluctuating ambulatory BP curve and the time axis using the composite trapezoid rule.^18^

Abbreviations: CI, confidence interval; DBP, diastolic blood pressure; Ref, reference; SBP, systolic blood pressure.

* *P*‐value were computed using generalized linear mix‐effect models with cluster‐specific random intercepts to account for the clustering effects within the data.

We also observed an overall proportion of 56.5% with ambulatory hypertension (24‐h ambulatory SBP/DBP ≥ 130/80 mmHg) (Table 2). Among the three countries, Bangladesh had the highest proportion of patients with ambulatory hypertension (72.6%, 95% confidence interval (CI) 63.5–80.2%), whereas lower proportions were observed in Sri Lanka (51.0%, 95% CI 41.3–60.7) and Pakistan (50.0%, 95% CI 42.7–57.3) (*P *= .014) (Figure 1B).

Moreover, the comparison of 24‐h cumulative SBP load demonstrated that Bangladesh had the highest SBP load (3231.1, SD 431.2), followed by Pakistan (2957.8, SD 437.6) and Sri Lanka (2950.2, SD 382.1) (*P *< .001) (Figure S2B).

### 3.3 Adjusted comparison of 24‐hour ambulatory, daytime, and nighttime SBP across countries

As shown in Table 3, after controlling for age, sex, clinic SBP, sociodemographic, lifestyle, comorbidities, medication usage, as well as clinical variables, the coefficient for 24‐h ambulatory SBP remained higher and statistically significant in Bangladesh (12.24, 95% CI 4.28 to 20.20) compared to Sri Lanka. Similarly, as shown in Tables 4 and 5, daytime SBP (11.96, 95% CI 3.87–20.06) and nighttime SBP (12.76, 95% CI 4.51–21.01) were consistently higher in Bangladesh than those in Sri Lanka. These differences with standardized effect size of 0.66 (95% CI 0.23–1.09) for 24‐h ambulatory SBP, 0.64 (95% CI 0.21–1.07) for daytime SBP, and 0.63 (95% CI 0.23–1.04) for nighttime SBP also suggested a large clinical significance.^22^ However, the levels of ambulatory SBP among patients in Pakistan were comparable to those in Sri Lanka.

**TABLE 3:** Patient characteristics associated with 24‐h ambulatory SBP among patients with uncontrolled clinic hypertension in rural Bangladesh, Pakistan, and Sri Lanka.

Characteristics	24‐h ambulatory SBP coefficient, mmHg (95% CI)	*P*‐value	Standardized coefficient (95% CI)
Country (Ref: Sri Lanka)		(−.005)	
Bangladesh	12.24 (4.28, 20.20)	.003	0.66 (0.23, 1.09)
Pakistan	7.39 (−2.04, 16.81)	.124	0.42 (−0.08, 0.92)
Age, per 1 year increase	0.13 (−0.05, 0.31)	.165	0.07 (−0.03, 0.18)
Male (Ref: Female)	3.99 (−1.14, 9.12)	.127	0.21 (−0.05, 0.48)
Clinic SBP, per 1 mmHg increase	0.38 (0.28, 0.47)	<.001	0.36 (0.26, 0.45)
**Sociodemographic variables**			
Educated (Ref: Not received formal education)	−2.90 (−7.40, 1.61)	.207	−0.15 (−0.38, 0.08)
Employed (Ref: currently unemployed)	−1.05 (−5.73, 3.62)	.658	−0.06 (−0.30, 0.19)
Socioeconomic levels (Ref: Poor)		(−.025)	
Middle	4.87 (0.16, 9.57)	.043	0.25 (0.01, 0.50)
Rich	9.02 (2.05, 15.98)	.011	0.46 (0.10, 0.82)
Married (Ref: Currently unmarried)	−4.81 (−9.06, −0.55)	.027	−0.25 (−0.47, −0.03)
**Lifestyle variables**			
Obese/overweight (Ref: Nonobese)	−1.75 (−5.52, 2.01)	.361	−0.09 (−0.29, 0.10)
Smoking habit (Ref: Never smoke)		(−.167)	
Former smoker	0.42 (−5.08, 5.92)	.881	0.02 (−0.27, 0.30)
Current smoker	5.86 (−0.77, 12.50)	.083	0.29 (−0.06, 0.63)
Frequency of fruit and vegetable intake per week, per 1 unit increase	−0.11 (−0.47, 0.25)	.543	−0.03 (−0.18, 0.12)
Physical activity level (Ref: Inactive)		(−.789)	
Minimally active	1.64 (−3.45, 6.73)	.526	0.08 (−0.18, 0.35)
Highly active	0.53 (−3.57, 4.64)	.799	0.03 (−0.18, 0.24)
**Comorbidities**			
Chronic kidney disease	4.94 (0.69, 9.19)	.023	0.28 (0.03, 0.52)
Diabetes	1.23 (−3.83, 6.29)	.631	0.05 (−0.22, 0.32)
**Medication usage**			
Number of antihypertensive medications, n (Ref = 0)		(−.763)	
1	2.38 (−4.15, 8.91)	.474	0.11 (−0.23, 0.45)
2	2.74 (−5.25, 10.74)	.500	0.12 (−0.29, 0.54)
3 or more	0.48 (−9.24, 10.20)	.923	0.01 (−0.50, 0.51)
Currently taking statins (Ref: Not currently taking statins)	3.91 (−1.91, 9.73)	.187	0.20 (−0.10, 0.50)
**Clinical variables**			
HDL cholesterol level, per 10 mg/dL increase	−1.37 (−3.26, 0.53)	.156	−0.07 (−0.20, 0.06)
LDL cholesterol level, per 10 mg/dL increase	0.12 (−0.41, 0.64)	.667	0.01 (−0.10, 0.12)
Triglycerides level, per 10 mg/dL increase	0.13 (−0.13, 0.39)	.311	0.06 (−0.06, 0.17)
(Intercept)	57.60 (33.41, 81.79)	<.001	−0.61 (−1.15, −0.07)

*Notes*: Obese/overweight is defined as BMI ≥ 23.5 kg/m^2^.^40^ Chronic kidney disease is defined as eGFR ≤ 60 mL/min per 1.73 m^2^ and/or urine albumin‐to‐creatinine ratio ≥ 30 mg/g.^41^ Diabetes mellitus is defined as fasting plasma glucose ≥ 126 mg/ml.^42^

Abbreviations: CI, confidence interval; HDL, high‐density lipoprotein; LDL, low‐density lipoprotein; SBP, systolic blood pressure.

*P*‐values in bold represented joint‐significance of all the categories using the likelihood‐based comparisons. The sample size for each country was as follows: Bangladesh (*N* = 106), Pakistan (*N* = 178), and Sri Lanka (*N* = 98).

**TABLE 4:** Patient characteristics associated with daytime SBP among patients with uncontrolled clinic hypertension in rural Bangladesh, Pakistan, and Sri Lanka.

Characteristics	Daytime SBP Coefficient, mmHg (95% CI)	*P‐*value	Standardized Coefficient (95% CI)
Country (Ref: Sri Lanka)		**(0.006)**	
Bangladesh	11.96 (3.87, 20.06)	0.004	0.64 (0.21, 1.07)
Pakistan	8.50 (−1.05, 18.06)	0.081	0.48 (−0.03, 0.98)
Age, per 1 year increase	0.08 (−0.11, 0.27)	0.396	0.04 (−0.06, 0.15)
Male (Ref: Female)	4.46 (−0.75, 9.67)	0.093	0.24 (−0.03, 0.51)
Clinic SBP, per 1 mmHg increase	0.37 (0.27, 0.47)	<0.001	0.35 (0.26, 0.44)
**Sociodemographic variables**			
Educated (Ref: Not received formal education)	−2.88 (−7.45, 1.69)	0.215	−0.15 (−0.38, 0.09)
Employed (Ref: currently unemployed)	−0.94 (−5.68, 3.80)	0.697	−0.05 (−0.30, 0.19)
Socioeconomic levels (Ref: Poor)		**(0.069)**	
Middle	4.26 (−0.51, 9.04)	0.080	0.22 (−0.03, 0.47)
Rich	7.69 (0.62, 14.76)	0.033	0.39 (0.02, 0.75)
Married (Ref: Currently unmarried)	−4.92 (−9.24, −0.60)	0.026	−0.26 (−0.48, −0.03)
**Lifestyle variables**			
Obese/overweight (Ref: Nonobese)	−2.22 (−6.06, 1.61)	0.255	−0.12 (−0.32, 0.08)
Smoking habit (Ref: Never smoke)		**(0.082)**	
Former smoker	−1.31 (−6.88, 4.27)	0.646	−0.07 (−0.36, 0.21)
Current smoker	6.32 (−0.40, 13.04)	0.065	0.31 (−0.04, 0.66)
Frequency of fruit and vegetable intake per week, per 1 unit increase	−0.08 (−0.44, 0.28)	0.651	−0.01 (−0.16, 0.14)
Physical activity level (Ref: Inactive)		**(0.685)**	
Minimally active	2.00 (−3.16, 7.16)	0.447	0.10 (−0.17, 0.37)
Highly active	1.28 (−2.88, 5.44)	0.545	0.07 (−0.15, 0.28)
**Comorbidities**			
Chronic kidney disease	4.32 (0.18, 8.46)	0.041	0.24 (0.00, 0.48)
Diabetes	0.80 (−4.13, 5.74)	0.749	0.03 (−0.23, 0.29)
**Medication usage**			
Number of antihypertensive medications, n (Ref = 0)		**(0.339)**	
1	4.43 (−2.19, 11.05)	0.189	0.21 (−0.13, 0.56)
2	4.44 (−3.65, 12.53)	0.281	0.21 (−0.21, 0.63)
3 or more	0.93 (−8.93, 10.79)	0.853	0.03 (−0.48, 0.54)
Currently taking statins (Ref: Not currently taking statins)	2.04 (−3.87, 7.95)	0.498	0.10 (−0.20, 0.41)
**Clinical variables**			
HDL cholesterol level, per 10 mg/dL increase	−1.07 (−3.01, 0.87)	0.276	−0.05 (−0.19, 0.08)
LDL cholesterol level, per 10 mg/dL increase	0.11 (−0.43, 0.65)	0.685	0.01 (−0.10, 0.12)
Triglycerides level, per 10 mg/dL increase	0.13 (−0.13, 0.39)	0.331	0.05 (−0.06, 0.17)
(Intercept)	63.48 (38.95, 88.01)	<0.001	−0.62 (−1.17, −0.08)

*Notes*: Obese/overweight is defined as BMI ≥ 23.5 kg/m^2^.^40^ Chronic kidney disease is defined as eGFR ≤ 60 mL/min per 1.73 m^2^ and/or urine albumin‐to‐creatinine ratio ≥ 30 mg/g.^41^ Diabetes mellitus is defined as fasting plasma glucose ≥ 126 mg/ml.^42^

Abbreviations: CI, confidence interval; HDL, high‐density lipoprotein, LDL, low‐density lipoprotein; SBP, systolic blood pressure.

*P*‐values in bold represented joint‐significance of all the categories using the likelihood‐based comparisons. The sample size for each country was as follows: Bangladesh (*N* = 106), Pakistan (*N* = 178), and Sri Lanka (*N* = 98).

**TABLE 5:** Patient characteristics associated with nighttime SBP among patients with uncontrolled clinic hypertension in rural Bangladesh, Pakistan, and Sri Lanka.

Characteristics	Nighttime SBP coefficient, mmHg (95% CI)	*P*‐value	Standardized coefficient (95% CI)
Country (Ref: Sri Lanka)		(.005)	
Bangladesh	12.76 (4.51, 21.01)	.003	0.63 (0.23, 1.04)
Pakistan	6.76 (−3.15, 16.67)	.180	0.35 (−0.12, 0.83)
Age, per 1 year increase	0.23 (0.02, 0.43)	.029	0.12 (0.01, 0.22)
Male (Ref: Female)	3.42 (−2.27, 9.12)	.238	0.17 (−0.10, 0.44)
Clinic SBP, per 1 mmHg increase	0.40 (0.29, 0.50)	<.001	0.34 (0.25, 0.44)
**Sociodemographic variables**			
Educated (Ref: Not received formal education)	−1.64 (−6.65, 3.37)	.521	−0.08 (−0.31, 0.16)
Employed (Ref: currently unemployed)	−0.52 (−5.69, 4.65)	.842	−0.02 (−0.27, 0.22)
Socioeconomic levels (Ref: Poor)		(.024)	
Middle	5.97 (0.78, 11.16)	.024	0.28 (0.04, 0.53)
Rich	9.57 (1.88, 17.26)	.015	0.45 (0.08, 0.81)
Married (Ref: Currently unmarried)	−5.15 (−9.89, −0.40)	.034	−0.25 (−0.47, −0.02)
**Lifestyle variables**			
Obese/overweight (Ref: Nonobese)	−1.09 (−5.25, 3.06)	.604	−0.05 (−0.25, 0.15)
Smoking habit (Ref: Never smoke)		(.447)	
Former smoker	2.55 (−3.56, 8.66)	.412	0.12 (−0.17, 0.41)
Current smoker	3.92 (−3.45, 11.29)	.296	0.17 (−0.18, 0.52)
Frequency of fruit and vegetable intake per week, per 1 unit increase	−0.19 (−0.56, 0.19)	.336	−0.05 (−0.20, 0.09)
Physical activity level (Ref: Inactive)		(.769)	
Minimally active	1.25 (−4.40, 6.90)	.664	0.06 (−0.21, 0.33)
Highly active	−0.56 (−5.11, 3.99)	.809	−0.02 (−0.24, 0.19)
**Comorbidities**			
Chronic kidney disease	6.09 (1.10, 11.08)	.017	0.31 (0.05, 0.57)
Diabetes	1.40 (−4.36, 7.16)	.631	0.05 (−0.23, 0.33)
**Medication usage**			
Number of antihypertensive medications, n (Ref = 0)		(.999)	
1	−0.48 (−7.63, 6.68)	.896	−0.04 (−0.37, 0.30)
2	0.01 (−8.79, 8.82)	.997	−0.02 (−0.43, 0.40)
3 or more	−0.41 (−11.14, 10.32)	.939	−0.04 (−0.54, 0.47)
Currently taking statins (Ref: Not currently taking statins)	6.98 (0.52, 13.43)	.034	0.33 (0.02, 0.63)
**Clinical variables**			
HDL cholesterol level, per 10 mg/dL increase	−1.84 (−3.89, 0.22)	.080	−0.09 (−0.22, 0.05)
LDL cholesterol level, per 10 mg/dL increase	0.07 (−0.50, 0.65)	.805	0.00 (−0.11, 0.11)
Triglycerides level, per 10 mg/dL increase	0.17 (−0.12, 0.45)	.254	0.06 (−0.05, 0.17)
(Intercept)	44.42 (17.76, 71.08)	.001	−0.59 (−1.12, −0.06)

*Notes*: Obese/overweight is defined as BMI ≥ 23.5 kg/m^2^.^40^ Chronic kidney disease is defined as eGFR ≤ 60 mL/min per 1.73 m^2^ and/or urine albumin‐to‐creatinine ratio ≥ 30 mg/g.^41^ Diabetes mellitus is defined as fasting plasma glucose ≥ 126 mg/ml.^42^

Abbreviations: CI, confidence interval; HDL, high‐density lipoprotein, LDL, low‐density lipoprotein; SBP, systolic blood pressure.

*P*‐values in bold represented joint‐significance of all the categories using the likelihood‐based comparisons. The sample size for each country was as follows: Bangladesh (*N* = 106), Pakistan (*N* = 178), and Sri Lanka (*N* = 98).

In addition, higher clinic SBP, currently unmarried (vs. married), and presence of CKD were significantly associated with elevated ambulatory SBP levels among patients with uncontrolled clinic hypertension (*P* < .05), even after adjusting for country, age, sex, and other patient covariates (Tables 3, 4, 5). Specifically, every 1 mmHg increase in clinic SBP was significantly associated with a rise in 24‐h ambulatory SBP (mean 0.38, 95% CI 0.28–0.47), daytime SBP (mean 0.37, 95% CI 0.27–0.47), and nighttime SBP (mean 0.40, 95% CI 0.29–0.50).

### 3.4 Sensitivity analysis

The results of the complete case analysis were consistent with those obtained from imputed datasets, except for marital status, which was no longer significantly associated with ambulatory SBP levels (Tables S2–S4).

In addition, the coefficient estimates of the country and patient characteristics remained robust after delta adjustment, indicating that our findings were unlikely to be substantially affected by the missing data mechanism (Figure S3).

Furthermore, the country‐level findings were consistent in terms of the magnitude or direction of the coefficients, indicating that there was no significant effect modification by patient characteristics in our study (Figure S4).

## 4 DISCUSSION

In 382 individuals with uncontrolled clinic hypertension from rural communities in Bangladesh, Pakistan, and Sri Lanka, we found significant cross‐country differences in the distribution of ABPM profiles among the three countries. Notably, individuals with uncontrolled clinic hypertension in Bangladesh exhibited a higher proportion of ambulatory hypertension and a higher level of 24‐h ambulatory SBP compared to those in Sri Lanka. Furthermore, this difference in 24‐h ambulatory SBP levels between the two countries persisted even after accounting for potential differences in patients’ sociodemographic, lifestyle, comorbidities, antihypertensive medication use, and clinical characteristics across the countries. Daytime and nighttime SBP exhibited similar patterns to 24‐h ambulatory SBP. Conversely, no significant differences were observed in the distribution of ABPM profiles between Sri Lanka and Pakistan. We also observed a high proportion of ambulatory hypertension and distinct variations between the distribution patterns of ambulatory SBP and clinic SBP levels. Moreover, our findings also indicated that higher clinic BP, being unmarried, and the presence of CKD were strong predictors of higher levels of ambulatory SBP in South Asia. However, these patient characteristics did not fully explain the observed cross‐country differences in ambulatory SBP levels. Our findings are the first to explore ABPM profiles and their cross‐country differences independent of individual‐level risk factors among rural populations in South Asia. This highlights the importance of incorporating 24‐h BP monitoring when evaluating and treating these patients with uncontrolled clinic hypertension, particularly in Bangladesh. Our findings also call for a more in‐depth analysis of the social determinants of health to understand the underlying causes for the cross‐country differences in ABPM profiles in South Asia.

It is important to note that while patients with uncontrolled clinic hypertension in Bangladesh had the highest proportion of ambulatory hypertension (72.6%), they were also most likely to use antihypertensive medications (98.1%) compared to the other two countries. Furthermore, patients with uncontrolled clinic hypertension in Bangladesh were also more likely to self‐report heart disease and stroke than their counterparts in Pakistan and Sri Lanka. This suggests a more unfavorable cardiovascular profile that could be linked to the poorer ABPM profiles of patients in Bangladesh. However, the possibility of reverse causality cannot be disregarded.

Consistent with previous studies, we identified several patient characteristics significantly associated with 24‐h ambulatory, daytime, and nighttime SBP. Notably, clinic SBP demonstrated a robust association with ambulatory SBP levels, even after adjusting for country, age, sex, and other potential covariates.^23^ In addition, in line with prior observations in this region,^24^, ^25^, ^26^, ^27^ being unmarried (vs. married) and the presence of CKD were also significantly associated with higher ambulatory SBP levels.

Interestingly, while patients in Bangladesh had significantly higher levels of 24‐h ambulatory, daytime, and nighttime SBP compared to Sri Lanka, no significant differences were observed in clinic SBP levels between the two countries. This finding was consistent with previous meta‐analyses that identified discrepancies in clinic BP versus ambulatory BP—with clinic BP measurements exhibiting approximately 74% accuracy compared to ABPM in identifying elevated BP.^28^, ^29^ Such discrepancy raises intriguing questions regarding the differences in the underlying factors and implications of clinic BP versus ambulatory BP levels. In accordance with prior research,^30^, ^31^, ^32^ our findings indicated that clinic SBP was not the only determinant of ambulatory SBP levels. This was evident from the weak correlation observed between clinic and ambulatory BP in several large observational studies involving both treated and untreated patients.^23^, ^33^ Other factors,^33^, ^34^ such as individual characteristics, cultural background, and environmental factors, collectively contributed to the elevation of ambulatory SBP levels, which have been identified as predictors of cardiovascular risk superior to clinic SBP.^10^, ^11^

In recent years, South Asia has undergone rapid demographic and economic transitions, which have contributed to an increase in hypertension‐related challenges. Our research findings have significant implications for public health in the region and support the broader use of ABPM in the clinical management of patients with uncontrolled clinic hypertension. Adopting ABPM can improve cardiovascular risk management, particularly for stratifying cardiovascular risk and for assessing antihypertensive treatment aimed at reducing major cardiovascular events.^35^ However, studies also show that adoption of ABPM was influenced by its high cost, as well as the acceptability of patients and healthcare providers. While studies in the United States and the UK have supported ABPM as the most cost‐effective strategy in diagnosing hypertension compared with clinic BP and home BP measurement,^36^, ^37^ evidence in LMIC settings is scarce. Therefore, there is a pressing need for future studies to evaluate the effectiveness and cost‐effectiveness of ABPM in LMIC settings to optimize its implementation and improve cardiovascular care for patients with uncontrolled clinic hypertension in resource‐constrained regions.

Our study also highlights that the vast majority of patients with uncontrolled clinic hypertension in Pakistan remained untreated, mainly due to the lack of free access to antihypertensive medications, which needs to be addressed urgently.

Our study has several strengths. It was the first to examine the distribution of ABPM profiles within a community‐based population in rural areas of Bangladesh, Pakistan, and Sri Lanka, providing novel insights into the cross‐country differences in ambulatory BP levels in patients with uncontrolled clinic hypertension in South Asia, which have remained understudied. We used standardized ABPM devices and implemented a common protocol across all countries, ensuring data consistency and comparability. By focusing on ABPM profiles and their associated factors, our study further aligned with the pressing public health challenges posed by the escalating CVD burden in South Asia, which holds the potential to guide subsequent studies and inform the design of targeted interventions.

Our study has some limitations. First, our population may not represent the entire population in the three countries. However, our study clusters were all randomly selected from their representative rural districts. Second, our population with a highly skewed distribution of patients taking antihypertensive medications across the three countries may introduce potential selection bias. Although the protocol was uniform in all three countries, a potential selection bias is possible as reflected in the varying use of antihypertensive medications among countries. However, this discrepancy may also reflect the actual gap in the proportions of patients with hypertension receiving antihypertensive medications across the three countries.^38^ Third, we acknowledged the possibility of overadjustment in our multivariable models, which could potentially underestimate true associations. Nevertheless, including these covariates was essential to capture the distinct characteristics of each country's population and healthcare system, ensuring a more thorough analysis of the cross‐country differences in ambulatory BP levels. Finally, the cross‐country differences in the distribution of ABPM profiles might be partially explained by other social and environmental determinants of health such as macroeconomic policies, social cohesion and networks, availability of green space, psychosocial stress, and the climate, which was not assessed in our study.^7^, ^39^ Larger, more representative studies with comprehensive measures of potential patient characteristics associated with ambulatory BP in South Asia are needed in the future to confirm our findings and better understand the reasons why patients with uncontrolled clinic hypertension in Bangladesh have higher levels of ambulatory SBP compared to those in Sri Lanka.

## 5 CONCLUSIONS

In conclusion, our findings revealed a high burden of ambulatory hypertension and cross‐country differences in the distribution of ABPM profile among the rural population with uncontrolled clinic hypertension in South Asia. Specifically, patients in Bangladesh had higher levels of 24‐h ambulatory, daytime, and nighttime SBP compared with those in Sri Lanka, indicating the need to incorporate 24‐h BP monitoring when evaluating and treating these patients to mitigate cardiovascular risk, particularly in Bangladesh. Our findings also identified several predictors of ambulatory SBP in South Asia, including higher clinic BP, being unmarried, and the presence of CKD. By exploring ABPM profiles in South Asia, our study underscored the need to confirm elevated clinic BP and consider additional predictors, such as ambulatory SBP levels, to enhance the management and prevention of hypertension‐related complications in this region.

## AUTHOR CONTRIBUTIONS

The authors’ contribution to the paper are outlines as follows: A. Zhu and T.H. Jafar conceptualized the idea. A. Zhu analyzed the data and wrote the paper, with input and contributions from T. Ostbye, M. Gandhi, and T.H. Jafar. A. Naheed, A. de Silva, and I. Jehan made equal contribution by collecting data for the COBRA trial as site principal investigator. N. Chakma, A. Kasturiratne, and Z. Samad curated the data for the COBRA trial. All authors approved the final version of the manuscript.

## CONFLICT OF INTEREST STATEMENT

The authors declare no conflicts of interest.

## PERMISSION TO REPRODUCE MATERIALS FROM OTHER SOURCES

None.

## CLINICAL TRIAL REGISTRATION

This work has been registered with clinicaltrials.gov (NCT02657746).

## Supporting information

Supporting Information

## Data Availability

The data supporting the findings of this study are available from the corresponding author T.H.J., tazeen.jafar@duke-nus.edu.sg, upon reasonable request.
