# Supplementary material for: Ambulatory blood pressure levels in individuals with uncontrolled clinic hypertension across Bangladesh, Pakistan, and Sri Lanka
Source: J Clin Hypertens (Greenwich). 2024 Mar 7;26(4):391–404. doi: 10.1111/jch.14787 (PMC11007786; doi:10.1111/jch.14787)
Supplement: Supplementary file 1 — Supporting Information [file JCH-26-391-s001.docx]

**Supplemental Material**

**Ambulatory Blood Pressure Levels in Individuals With Uncontrolled Clinic Hypertension Across Bangladesh, Pakistan, And Sri Lanka**

Anqi Zhu, BS^a^; Truls Ostbye, MD MPH^b^; Aliya Naheed, MBBS MPH PHD^c*^; Asita de Silva, MBBS DPhil FRCP^d*^; Imtiaz Jehan, MBBS MSc FCPS^e*^; Mihir Gandhi, BSc MSc PhD CStat^f^; Nantu Chakma, BSS MSSc^c^; Anuradhani Kasturiratne, MBBS MSc MD^g^; Zainab Samad, MBBS MHS^h^; Tazeen Hasan Jafar, MD MPH^a, i^

^a^Program in Health Services & Systems Research, Duke-NUS Medical School, Singapore.

^b^Duke University Department of Family Medicine and Community Health, Duke University Medical Center, Durham, USA.

^c^Health Systems and Population Studies Division, International Centre for Diarrhoeal Disease Research (ICDDR, B), Dhaka, Bangladesh.

^d^Department of Pharmacology, Faculty of Medicine, University of Kelaniya, Ragama, Sri Lanka.

^e^Department of Community Health Science, Aga Khan University, Karachi, Pakistan.

^f^Singapore Clinical Research Institute, Consortium for Clinical Research and Innovation Singapore, Singapore; Centre of Quantitative Medicine, Duke-NUS Medical School, Singapore; and Tampere Center for Child Health Research, Tampere University, Tampere, Finland.

^g^Department of Public Health, Faculty of Medicine, University of Kelaniya, Ragama, Sri Lanka.

^h^Department of Medicine, Medical College, Aga Khan University, Karachi, Pakistan.

^i^Duke Global Health Institute, Durham, NC, USA.

*contributed equally to data

**Corresponding Author:** Prof Tazeen H. Jafar

Supplemental Methods

**Covariates**:

We considered several potential patient characteristics as covariates associated with ambulatory blood pressure monitoring (ABPM) profile based on the previous literature and variable availability. Sociodemographic variables included age, sex, marital status (currently unmarried vs married), education status (no formal education vs educated), employment status (currently unemployed vs. employed), and socioeconomic level.^1,2^ To measure socioeconomic level, we calculated the international wealth index by adding a constant to a weighted sum of household facilities indicators, including television, refrigerator, phone, car, bicycle, utensils, floor quality, toilet quality, sleeping rooms, electricity, and water quality.^3^ Patients were then classified into socioeconomic levels based on their international wealth index scores within their respective country group: poor (bottom 15%), middle (15%-85%), and rich (top 15%).^4^

In addition, patients reported their medication usage including statins usage (currently using statins vs. currently not on statins) and the number of antihypertensive medication usage (0, 1, 2, ≥ 3), smoking habits (never smoke, former smoker, current smoker), and physical activity level assessed using the International Physical Activity Questionnaire (inactive, minimally active, highly active).^5^ Combined frequency of fruit and vegetable intake per week was obtained via an abbreviated food frequency questionnaire.^6^

Weight and height were measured and converted to body mass index (BMI). Patients were classified as obese/overweight if their BMI was over 23.5 kg/m^2^.^7^ Patients’ estimated glomerular filtration rate (eGFR), urine spot albumin-to-creatinine ratio (ACR), total cholesterol, high-density lipoprotein cholesterol (HDL), low-density lipoprotein cholesterol (LDL), triglycerides, serum creatinine, and fasting plasma glucose were collected through standard lab results of blood and urine. Chronic kidney disease (CKD) was defined as eGFR ≤ 60 ml/min/1.73m^2^ and/or urine ACR ≥ 30 mg/g^8^, and diabetes mellitus was defined as fasting plasma glucose ≥ 126 mg/dL.^9^

**Supplemental Tables**

Table S1 Distributions of Observed and Imputed Data

| Variables | N missing (%) | Observed | Imputed |
| --- | --- | --- | --- |
|  |  | mean (SD) for continuous or n (%) for categorical variables | mean (SD) for continuous or n (%) for categorical variables |
| Outcomes |  |  |  |
| 24-hour ambulatory SBP, mmHg | 0 (0.0%) | 131.8 (19.3) | NA |
| Nighttime SBP, mmHg | 0 (0.0%) | 80.6 (11.1) | NA |
| Patient Characteristics | | | |
| Age, year | 0 (0.0%) | 58.3 (11.0) | NA |
| Male (Ref: Female) | 0 (0.0%) | 135 (35.3%) | NA |
| Country | 0 (0.0%) |  |  |
| Bangladesh |  | 106 (27.7%) | NA |
| Pakistan |  | 178 (46.6%) | NA |
| Sri Lanka |  | 98 (25.7%) | NA |
| Married (Ref: Currently unmarried) | 0 (0.0%) | 280 (73.3%) | NA |
| Socioeconomic level | 0 (0.0%) |  |  |
| Poor |  | 73 (19.1%) | NA |
| Middle |  | 264 (69.1%) | NA |
| Rich |  | 45 (11.8%) | NA |
| Educated (Ref: Not received formal education) | 0 (0.0%) | 198 (51.8%) | NA |
| Employed (Ref: Currently unemployed) | 0 (0.0%) | 114 (29.8%) | NA |
| Obese/overweight (Ref: Nonobese) | 3 (0.8%) | 221 (58.3%) | 222 (58.1%) |
| Frequency of fruit and vegetable intake per week | 6 (1.6%) | 12.5 (8.0) | 12.5 (8.0) |
| Physical activity level | 2 (0.5%) |  |  |
| Inactive |  | 115 (30.3%) | 115 (30.1%) |
| Minimally active |  | 73 (19.2%) | 74 (19.4%) |
| Highly active |  | 192 (50.5%) | 193 (50.5%) |
| Smoking habit | 0 (0.0%) |  |  |
| Never smoke |  | 294 (77.0%) | NA |
| Former smoker |  | 57 (14.9%) | NA |
| Current smoker |  | 31 (8.1%) | NA |
| Self-reported heart disease | 0 (0.0%) | 39 (10.2%) | NA |
| Chronic kidney disease | 29 (7.6%) | 165 (43.2%) | 178 (46.6%) |
| Diabetes | 23 (6.0%) | 76 (21.2%) | 89 (23.3%) |
| Self-reported stroke | 0 (0.0%) | 42 (11.0%) | NA |
| Clinic SBP, mmHg | 0 (0.0%) | 157.5 (18.2) | NA |
| Number of antihypertensive medications used | 0 (0.0%) |  |  |
| 0 |  | 156 (40.8%) | NA |
| 1 |  | 135 (35.3%) | NA |
| 2 |  | 69 (18.1%) | NA |
| 3 or more |  | 22 (5.8%) | NA |
| Currently taking satins (Ref: Not currently taking statins) | 0 (0.0%) | 52 (13.6%) | NA |
| HDL cholesterol, mg/dL | 24 (6.3%) | 44.5 (12.4) | 44.6 (12.4) |
| LDL cholesterol, mg/dL | 25 (6.5%) | 122.5 (37.6) | 122.5 (37.5) |
| Triglycerides, mg/dL | 25 (6.5%) | 149.9 (75.1) | 149.0 (74.3) |

**Abbreviations**: SD = standard deviation; SBP = systolic blood pressure; HDL = high-density lipoprotein; LDL = low-density lipoprotein.

Obese /Overweight is defined as BMI ≥ 23.5 kg/m^2^ ; Chronic kidney disease is defined as eGFR (estimated glomerular filtration rate) ≤ 60 mL/minute per 1.73 m^2^ and/or urine albumin-to-creatinine ratio ≥ 30 mg/g; Diabetes mellitus is defined as fasting plasma glucose ≥ 126 mg/ml.

Incomplete variables were imputed using fully conditional two-level multiple imputation methods, with country as the level-2 variable. Continuous and polynomial variables were imputed based on the predictive mean matching (PMM) method, whereas binary variables were imputed using logistic regression.

Table S2 Complete-case Analysis of Characteristics associated with 24-hour Ambulatory SBP among Patients with Uncontrolled Clinic Hypertension in Rural Bangladesh, Pakistan, and Sri Lanka

| Characteristics | 24-hour Ambulatory SBP Coefficient, mmHg  (95% CI) | *P*-value | Standardized Coefficient  ( 95% CI) |
| --- | --- | --- | --- |
| Country (Ref: Sri Lanka) |  | **(0.013)** |  |
| Bangladesh | 11.82 (4.24, 19.39) | 0.007 | 0.61 (0.22, 1.00) |
| Pakistan | 6.95 (-2.31, 16.01) | 0.168 | 0.36 (-0.12, 0.83) |
| Age, per 1 year increase | 0.21 (0.02, 0.39) | 0.037 | 0.12 (0.01, 0.22) |
| Male (Ref: Female) | 3.29 (-2.02, 8.70) | 0.246 | 0.17 (-0.10, 0.45) |
| Clinic SBP, per 1 mmHg increase | 0.40 (0.30, 0.50) | <0.001 | 0.38 (0.28, 0.47) |
| **Sociodemographic variables** | | | |
| Educated (Ref: Not received formal education) | -2.55 (-6.88, 1.85) | 0.269 | -0.13 (-0.36, 0.10) |
| Employed (Ref: currently unemployed) | 0.46 (-4.12, 5.42) | 0.855 | 0.02 (-0.21, 0.28) |
| Socioeconomic levels (Ref: Poor) |  | **(0.016)** |  |
| Middle | 6.45 (1.56, 11.27) | 0.013 | 0.33 (0.08, 0.58) |
| Rich | 9.56 (2.42, 16.87) | 0.013 | 0.50 (0.13, 0.87) |
| Married (Ref: Currently unmarried) | -3.80 (-8.36, 0.45) | 0.099 | -0.20 (-0.43, 0.02) |
| **Lifestyle variables** |  |  |  |
| Obese/overweight (Ref: Nonobese) | -2.33 (-6.14, 1.37) | 0.240 | -0.12 (-0.32, 0.07) |
| Smoking habit (Ref: Never smoke) |  | **(0.439)** |  |
| Former smoker | -1.47 (-7.02, 4.07) | 0.616 | -0.08 (-0.36, 0.21) |
| Current smoker | 3.15 (-3.57, 10.23) | 0.386 | 0.16 (-0.19, 0.53) |
| Frequency of fruit and vegetable intake per week, per 1 unit increase | -0.17 (-0.61, 0.20) | 0.357 | -0.07 (-0.25, 0.08) |
| Physical activity level (Ref: Inactive) |  | **(0.639)** |  |
| Minimally active | 2.19 (-2.76, 7.27) | 0.408 | 0.11 (-0.14, 0.38) |
| Highly active | 0.17 (-3.80, 4.38) | 0.937 | 0.01 (-0.20, 0.23) |
| **Comorbidities** |  |  |  |
| Chronic kidney disease | 5.63 (1.57, 9.52) | 0.008 | 0.29 (0.08, 0.49) |
| Diabetes | 2.05 (-2.30, 6.29) | 0.365 | 0.11 (-0.12, 0.33) |
| **Medication usage** |  |  |  |
| Number of antihypertensive medications, n (Ref = 0) |  | **(0.856)** |  |
| 1 | 1.46 (-4.78, 8.72) | 0.669 | 0.08 (-0.25, 0.45) |
| 2 | 0.58 (-7.06, 9.21) | 0.889 | 0.03 (-0.37, 0.48) |
| 3 or more | -0.90 (-9.94, 8.94) | 0.856 | -0.05 (-0.52, 0.46) |
| Currently taking statins (Ref: Not currently taking statins) | 4.31 (-1.40, 10.36) | 0.164 | 0.22 (-0.07, 0.54) |
| **Clinical variables** |  |  |  |
| HDL cholesterol level, per 10 mg/dL increase | -1.57 (-3.36, 0.21) | 0.096 | -0.10 (-0.22, 0.01) |
| LDL cholesterol level, per 10 mg/dL increase | 0.20 (-0.31, 0.72) | 0.468 | 0.04 (-0.06, 0.14) |
| Triglycerides level, per 10 mg/dL increase | 0.04 (-0.21, 0.29) | 0.734 | 0.02 (-0.08, 0.11) |
| (Intercept) | 50.47 (27.38, 75.95) | <0.001 | -0.63 (-1.16, -0.11) |

**Abbreviations**: CI = confidence interval; SBP = systolic blood pressure; HDL = high-density lipoprotein, LDL = low-density lipoprotein.

Obese /Overweight is defined as BMI ≥ 23.5 kg/m^2^; Chronic kidney disease is defined as eGFR (estimated glomerular filtration rate) ≤ 60 mL/minute per 1.73 m^2^ and/or urine albumin-to-creatinine ratio ≥ 30 mg/g; Diabetes mellitus is defined as fasting plasma glucose ≥ 126 mg/ml.

P-values in bold represented joint-significance of all the categories. The sample size for each country were as follows: Bangladesh (N = 103), Pakistan (N = 151), and Sri Lanka (N = 85).

Table S3 Complete-case Analysis of Characteristics associated with Daytime SBP among Patients with Uncontrolled Clinic Hypertension in Rural Bangladesh, Pakistan, and Sri Lanka

| Characteristics | Daytime SBP Coefficient, mmHg (95%CI) | *P*-value | Standardized Coefficients (95%CI) |
| --- | --- | --- | --- |
| Country (Ref: Sri Lanka) |  | **(0.017)** |  |
| Bangladesh | 11.69 (3.82, 19.55) | 0.010 | 0.61 (0.22, 1.00) |
| Pakistan | 8.35 (-1.19, 17.73) | 0.110 | 0.36 (-0.12, 0.83) |
| Age, per 1 year increase | 0.16 (-0.03, 0.35) | 0.111 | 0.12 (0.01, 0.22) |
| Male (Ref: Female) | 4.02 (-1.38, 9.52) | 0.164 | 0.17 (-0.10, 0.45) |
| Clinic SBP, per 1 mmHg increase | 0.39 (0.29, 0.49) | <0.001 | 0.38 (0.28, 0.47) |
| **Sociodemographic variables** | |  |  |
| Educated (Ref: Not received formal education) | -2.36 (-6.82, 2.06) | 0.314 | -0.13 (-0.36, 0.10) |
| Employed (Ref: currently unemployed) | 0.70 (-3.96, 5.75) | 0.783 | 0.02 (-0.21, 0.28) |
| Socioeconomic levels (Ref: Poor) |  | **(0.060)** |  |
| Middle | 5.55 (0.61, 10.50) | 0.035 | 0.33 (0.08, 0.58) |
| Rich | 7.77 (0.52, 15.28) | 0.048 | 0.50 (0.13, 0.87) |
| Married (Ref: Currently unmarried) | -4.02 (-8.66, 0.29) | 0.086 | -0.20 (-0.43, 0.02) |
| **Lifestyle variables** |  |  |  |
| Obese/overweight (Ref: Nonobese) | -2.75 (-6.59, 1.05) | 0.173 | -0.12 (-0.32, 0.07) |
| Smoking habit (Ref: Never smoke) |  | **(0.167)** |  |
| Former smoker | -3.62 (-9.25, 2.04) | 0.225 | -0.08 (-0.36, 0.21) |
| Current smoker | 3.21 (-3.64, 10.39) | 0.384 | 0.16 (-0.19, 0.53) |
| Frequency of fruit and vegetable intake per week, per 1 unit increase | -0.12 (-0.57, 0.26) | 0.517 | -0.07 (-0.25, 0.08) |
| Physical activity level (Ref: Inactive) |  | **(0.667)** |  |
| Minimally active | 2.29 (-2.76, 7.43) | 0.395 | 0.11 (-0.14, 0.38) |
| Highly active | 0.86 (-3.18, 5.14) | 0.695 | 0.01 (-0.20, 0.23) |
| **Comorbidities** |  |  |  |
| Chronic kidney disease | 4.98 (0.87, 8.95) | 0.020 | 0.29 (0.08, 0.49) |
| Diabetes | 1.49 (-2.94, 5.79) | 0.518 | 0.11 (-0.12, 0.33) |
| **Medication usage** |  |  |  |
| Number of antihypertensive medications, n (Ref = 0) |  | **(0.474)** |  |
| 1 | 3.58 (-2.82, 11.19) | 0.305 | 0.08 (-0.25, 0.45) |
| 2 | 2.02 (-5.78, 11.02) | 0.637 | 0.03 (-0.37, 0.48) |
| 3 or more | -0.41 (-9.62, 9.72) | 0.934 | -0.05 (-0.52, 0.46) |
| Currently taking statins (Ref: Not currently taking statins) | 2.70 (-3.11, 8.86) | 0.390 | 0.22 (-0.07, 0.54) |
| **Clinical variables** |  |  |  |
| HDL cholesterol level, per 10 mg/dL increase | -1.30 (-3.12, 0.51) | 0.178 | -0.10 (-0.22, 0.01) |
| LDL cholesterol level, per 10 mg/dL increase | 0.19 (-0.33, 0.72) | 0.503 | 0.04 (-0.06, 0.14) |
| Triglycerides level, per 10 mg/dL increase | 0.05 (-0.21, 0.30) | 0.709 | 0.02 (-0.08, 0.11) |
| (Intercept) | 55.88 (32.32, 81.78) | <0.001 | -0.63 (-1.16, -0.11) |

**Abbreviations**: CI = confidence interval; SBP = systolic blood pressure; HDL = high-density lipoprotein, LDL = low-density lipoprotein.

Obese /Overweight is defined as BMI ≥ 23.5 kg/m^2^; Chronic kidney disease is defined as eGFR (estimated glomerular filtration rate) ≤ 60 mL/minute per 1.73 m^2^ and/or urine albumin-to-creatinine ratio ≥ 30 mg/g; Diabetes mellitus is defined as fasting plasma glucose ≥ 126 mg/ml.

P-values in bold represented joint-significance of all the categories using the likelihood-based comparisons. The sample size for each country were as follows: Bangladesh (N = 103), Pakistan (N = 151), and Sri Lanka (N = 85).

Table S4 Complete-case Analysis of Characteristics associated with Nighttime SBP among Patients with Uncontrolled Clinic Hypertension in Rural Bangladesh, Pakistan, and Sri Lanka

| Characteristics | Nighttime SBP Coefficient, mmHg  (95% CI) | *P*-value | Standardized Coefficient  (95% CI) |
| --- | --- | --- | --- |
| Country (Ref: Sri Lanka) |  | **(0.009)** |  |
| Bangladesh | 12.31 (4.71, 19.98) | 0.005 | 0.58 (0.22, 0.95) |
| Pakistan | 6.11 (-3.46, 15.38) | 0.239 | 0.29 (-0.16, 0.73) |
| Age, per 1 year increase | 0.29 (0.09, 0.51) | 0.008 | 0.15 (0.05, 0.26) |
| Male (Ref: Female) | 2.00 (-3.97, 7.97) | 0.528 | 0.09 (-0.19, 0.38) |
| Clinic SBP, per 1 mmHg increase | 0.42 (0.30, 0.52) | <0.001 | 0.36 (0.26, 0.45) |
| **Sociodemographic variables** | | | |
| Educated (Ref: Not received formal education) | -1.44 (-6.22, 3.62) | 0.578 | -0.07 (-0.29, 0.17) |
| Employed (Ref: currently unemployed) | 0.84 (-4.22, 6.47) | 0.764 | 0.04 (-0.20, 0.31) |
| Socioeconomic levels (Ref: Poor) |  | **(0.009)** |  |
| Middle | 7.96 (2.50, 13.22) | 0.006 | 0.38 (0.12, 0.63) |
| Rich | 10.78 (2.84, 18.77) | 0.012 | 0.51 (0.13, 0.89) |
| Married (Ref: Currently unmarried) | -4.02 (-9.14, 0.75) | 0.121 | -0.19 (-0.43, 0.04) |
| **Lifestyle variables** |  |  |  |
| Obese/overweight (Ref: Nonobese) | -1.77 (-6.11, 2.33) | 0.427 | -0.08 (-0.29, 0.11) |
| Smoking habit (Ref: Never smoke) |  | **(0.813)** |  |
| Former smoker | 1.52 (-4.73, 7.67) | 0.644 | 0.07 (-0.22, 0.36) |
| Current smoker | 1.93 (-5.51, 10.01) | 0.636 | 0.09 (-0.26, 0.47) |
| Frequency of fruit and vegetable intake per week, per 1 unit increase | -0.28 (-0.70, 0.09) | 0.157 | -0.10 (-0.26, 0.03) |
| Physical activity level (Ref: Inactive) |  | **(0.569)** |  |
| Minimally active | 2.32 (-3.22, 8.06) | 0.435 | 0.11 (-0.15, 0.38) |
| Highly active | -0.46 (-4.88, 4.23) | 0.849 | -0.02 (-0.23, 0.2) |
| **Comorbidities** |  |  |  |
| Chronic kidney disease | 6.83 (2.23, 11.17) | 0.004 | 0.32 (0.11, 0.53) |
| Diabetes | 2.43 (-2.43, 7.22) | 0.341 | 0.11 (-0.12, 0.34) |
| **Medication usage** |  |  |  |
| Number of antihypertensive medications, n (Ref = 0) |  | **(0.992)** |  |
| 1 | -1.43 (-8.19, 6.08) | 0.703 | -0.07 (-0.39, 0.29) |
| 2 | -1.52 (-9.93, 7.59) | 0.744 | -0.07 (-0.47, 0.36) |
| 3 or more | -1.83 (-11.90, 8.76) | 0.738 | -0.09 (-0.56, 0.41) |
| Currently taking statins (Ref: Not currently taking statins) | 6.97 (0.61, 13.72) | 0.045 | 0.33 (0.03, 0.65) |
| **Clinical variables** |  |  |  |
| HDL cholesterol level, per 10 mg/dL increase | -2.02 (-4.01, -0.03) | 0.056 | -0.12 (-0.24, 0.00) |
| LDL cholesterol level, per 10 mg/dL increase | 0.15 (-0.43, 0.73) | 0.613 | 0.03 (-0.08, 0.13) |
| Triglycerides level, per 10 mg/dL increase | 0.08 (-0.20, 0.36) | 0.598 | 0.03 (-0.07, 0.13) |
| (Intercept) | 38.71 (13.11, 66.51) | 0.006 | -0.62 (-1.13, -0.11) |

**Abbreviations**: CI = confidence interval; SBP = systolic blood pressure; HDL = high-density lipoprotein, LDL = low-density lipoprotein.

Obese /Overweight is defined as BMI ≥ 23.5 kg/m^2^; Chronic kidney disease is defined as eGFR (estimated glomerular filtration rate) ≤ 60 mL/minute per 1.73 m^2^ and/or urine albumin-to-creatinine ratio ≥ 30 mg/g; Diabetes mellitus is defined as fasting plasma glucose ≥ 126 mg/ml.

P-values in bold represented joint-significance of all the categories using the likelihood-based comparisons. The sample size for each country were as follows: Bangladesh (N = 103), Pakistan (N = 151), and Sri Lanka (N = 85).

**Supplemental Figures**

Figure S1 Trends of Cardiovascular Disease Burden in South Asia and Stroke-specific Age-Standardized Disability-Adjusted Life Years

1. Bar plots showing the overall burden of cardiovascular disease in South Asia as disability-adjusted life years (DALY) from 1990 to 2019. Pink indicates the fraction of those DALYs attributable to stroke. B. Comparison of age-standardized DALYs for stroke per 100,000 people across Bangladesh (orange), Pakistan (blue), and Sri Lanka (grey) in 2019, accompanied by error bars representing the 95% confidence interval (CI). Data source: Global Burden of Disease (GBD) 2019 Results, the Institute for Health Metrics and Evaluation (IHME), 2023.^10^

Figure S2 Comparison of the Distributions of Clinic Systolic Blood Pressure and Ambulatory Blood Pressure Monitoring Profiles among Patients with Uncontrolled Clinic Hypertension across Rural Bangladesh, Pakistan, and Sri Lanka.

A. Violin plots comparing the distribution of clinic systolic blood pressure (SBP) (pink) , 24-hour ambulatory SBP (grey), daytime SBP (orange), and nighttime SBP (blue) levels in patients with uncontrolled clinic hypertension across Bangladesh, Pakistan, and Sri Lanka. B. Violin plots comparing the distribution of 24-hour cumulative SBP load across patients in the three countries. The 24-hour cumulative SBP and DBP load were calculated as the area under curve (AUC) between fluctuating ambulatory BP curve and the time axis using the composite trapezoid rule.^11^ The black dots represented the mean and error bars represented the 95% confidence interval (CI). Pairwise comparison was conducted using the t-test, with Sri Lanka serving as the reference group. ns ≥ 0.05, * p<0.05, ** p<0.001.

**
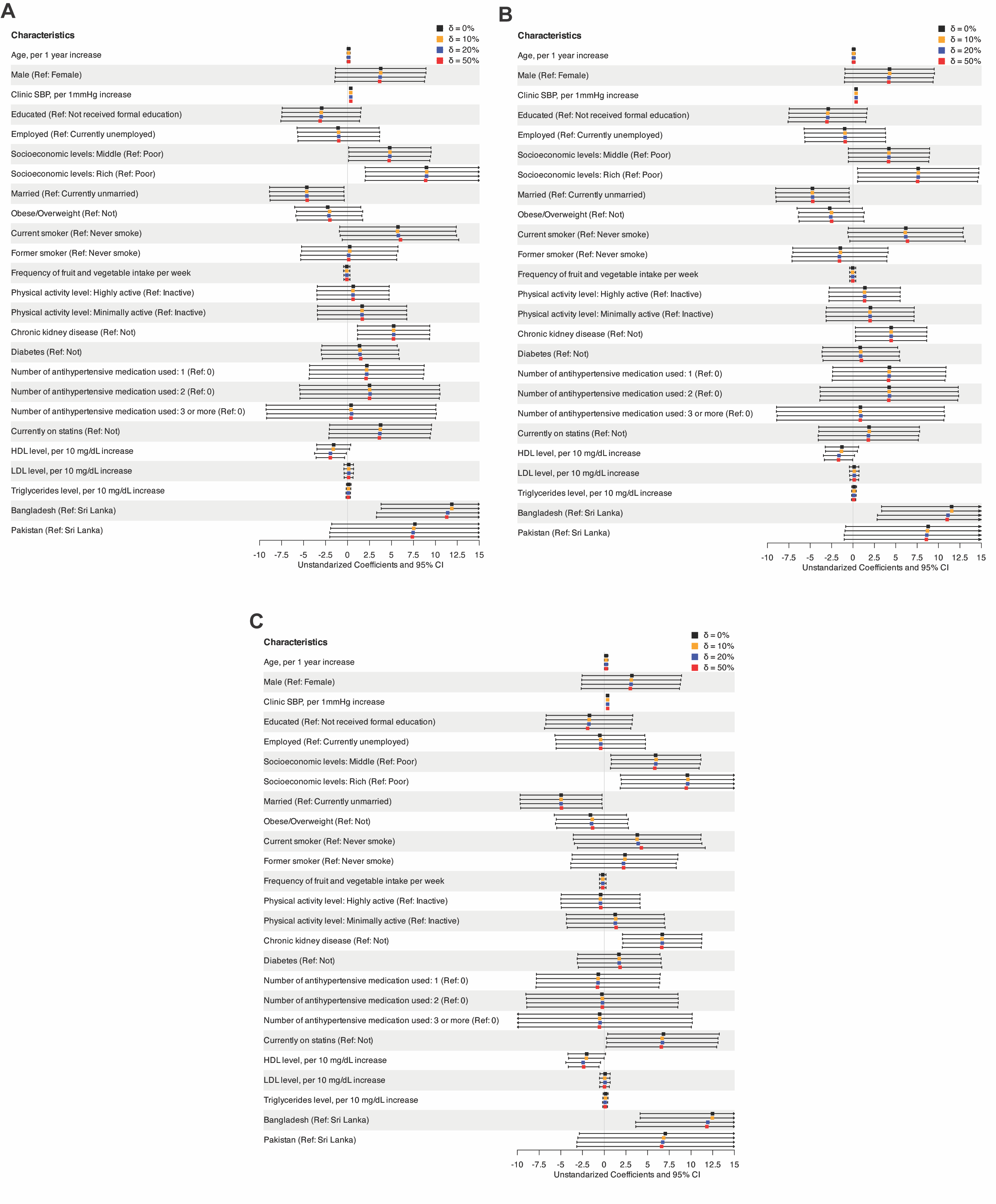
**

**Figure S3 Forest Plot of Multivariable Regression Coefficients for 24-hour Ambulatory, Daytime, and Nighttime Systolic Blood Pressure after Delta Adjustment**.

**A.** Multivariable models for 24-hour ambulatory systolic blood pressure (SBP) were built with multiple imputed datasets after delta-adjustment at δ = 0%, 10%, 20%, and 50% of the means of the observed continuous data. Similarly, multivariable models for daytime SBP (**B**) and nighttime SBP (**C**) were also built with multiple imputed datasets after delta-adjustment at δ = 0%, 10%, 20%, and 50% of the means of the observed continuous data. Colour black, orange, blue, and red indicated results with δ = 0%, 10%, 20%, and 50%, respectively. HDL indicates high-density lipoprotein; LDL, low-density lipoprotein.

**
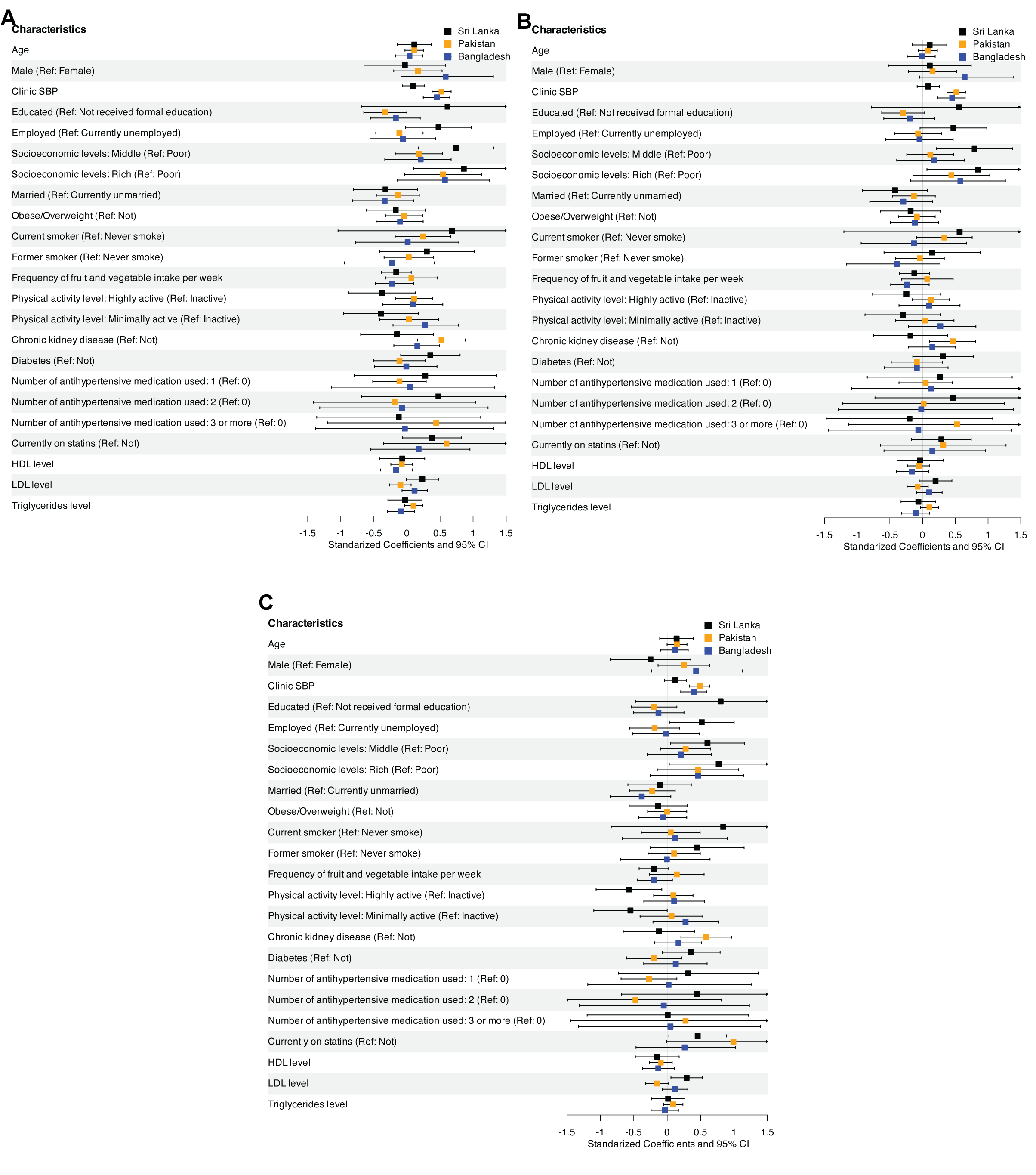
**

**Figure S4 Forest Plot of Multivariable Models for 24-hour Ambulatory, Daytime, and Nighttime Systolic Blood Pressure, Stratified by Country.**

**A.** Stratified analysis of standardized coefficients for 24-hour ambulatory systolic blood pressure (SBP) by country. Similarly, **s**tratified analysis of standardized coefficients for daytime SBP (**B**) and nighttime SBP (**C**) by country. Colour black, orange, and blue indicated models built in country Sri Lanka, Pakistan, and Bangladesh, respectively. HDL indicates high-density lipoprotein; LDL, low-density lipoprotein.

**Reference:**

1. Katulanda P, Ranasinghe P, Jayawardena R, Constantine GR, Rezvi Sheriff MH, Matthews DR. The prevalence, predictors and associations of hypertension in Sri Lanka: a cross-sectional population based national survey. *Clin Exp Hypertens*. 2014;36(7):484-91. doi:10.3109/10641963.2013.863321

2. Rahman MM, Gilmour S, Akter S, Abe SK, Saito E, Shibuya K. Prevalence and control of hypertension in Bangladesh: a multilevel analysis of a nationwide population-based survey. *J Hypertens*. Mar 2015;33(3):465-72; discussion 472. doi:10.1097/HJH.0000000000000421

3. Smits J. Ethnic intermarriage and social cohesion. What can we learn from Yugoslavia? *Social indicators research*. May 2010;96(3):417-432. doi:10.1007/s11205-009-9485-y

4. Gandhi M, Assam PN, Turner EL, et al. Statistical analysis plan for the control of blood pressure and risk attenuation-rural Bangladesh, Pakistan, Sri Lanka (COBRA-BPS) trial: a cluster randomized trial for a multicomponent intervention versus usual care in hypertensive patients. *Trials*. Nov 29 2018;19(1):658. doi:10.1186/s13063-018-3022-8

5. Ranasinghe CD, Ranasinghe P, Jayawardena R, Misra A. Physical activity patterns among South-Asian adults: a systematic review. *International Journal of Behavioral Nutrition and Physical Activity*. Oct 12 2013;10(116):1-11. doi:10.1186/1479-5868-10-116

6. Jafar TH, Silva A, Naheed A, et al. Control of blood pressure and risk attenuation: a public health intervention in rural Bangladesh, Pakistan, and Sri Lanka: feasibility trial results. *J Hypertens*. Sep 2016;34(9):1872-81. doi:10.1097/HJH.0000000000001014

7. WHO Expert Consultation. Appropriate body-mass index for Asian populations and its implications for policy and intervention strategies. *Lancet*. Mar 2004;363(9403):157-163. doi:10.1016/S0140-6736(03)15268-3

8. Levin A, Stevens PE, Bilous RW, et al. Kidney Disease: Improving Global Outcomes (KDIGO) CKD Work Group. KDIGO 2012 clinical practice guideline for the evaluation and management of chronic kidney disease. *Kidney international supplements*. 2013;3(1):1-150. doi:10.1038/kisup.2012.73

9. American Diabetes Association. Diagnosis and classification of diabetes mellitus. *Diabetes care*. 2014;37(Supplement_1):S81-S90. doi:10.2337/dc14-S081

10. Global Burden of Disease Study 2019 (GBD 2019). Seattle, United States: Institute for Health Metrics and Evaluation (IHME), 2020. (Last Accessed Dec 13, 2023). <https://vizhub.healthdata.org/gbd-results/>

11. Zhou B, Li C, Shou J, Zhang Y, Wen C, Zeng C. The cumulative blood pressure load and target organ damage in patients with essential hypertension. *J Clin Hypertens (Greenwich)*. Jun 2020;22(6):981-990. doi:10.1111/jch.13875
